# Orf1B controls secretion of T3SS proteins and contributes to *Edwardsiella piscicida* adhesion to epithelial cells

**DOI:** 10.1186/s13567-022-01057-6

**Published:** 2022-06-13

**Authors:** Long Kun Wang, Shan Shan Sun, Shu Ya Zhang, Pin Nie, Hai Xia Xie

**Affiliations:** 1 Dalian Ocean University, Dalian, 116023 Liaoning China; 2 State Key Laboratory of Freshwater Ecology and Biotechnology, Institute of Hydrobiology, Chinese Academy of Sciences, Wuhan, 430072 Hubei China; 3 College of Advanced Agricultural Sciences, University of Chinese Academy of Sciences, Beijing, 101408 China

**Keywords:** T3SS, Orf1B, adhesion, T3SS protein secretion, *Edwardsiella piscicida*

## Abstract

*Edwardsiella piscicida* is a Gram-negative enteric pathogen that causes hemorrhagic septicemia in fish. The type III secretion system (T3SS) is one of its two most important virulence islands. T3SS protein EseJ inhibits *E. piscicida* adhesion to epithelioma papillosum cyprini (EPC) cells by negatively regulating type 1 fimbria. Type 1 fimbria helps *E. piscicida* to adhere to fish epithelial cells. In this study, we characterized a functional unknown protein (Orf1B) encoded within the T3SS gene cluster of *E. piscicida*. This protein consists of 122 amino acids, sharing structural similarity with YscO in *Vibrio parahaemolyticus*. Orf1B controls secretion of T3SS translocon and effectors in *E. piscicida*. By immunoprecipitation, Orf1B was shown to interact with T3SS ATPase EsaN. This interaction may contribute to the assembly of the ATPase complex, which energizes the secretion of T3SS proteins. Moreover, disruption of Orf1B dramatically decreased *E. piscicida* adhesion to EPC cells due to the increased steady-state protein level of EseJ within *E. piscicida*. Taken together, this study partially unraveled the mechanisms through which Orf1B promotes secretion of T3SS proteins and contributes to *E. piscicida* adhesion. This study helps to improve our understanding on molecular mechanism of *E. piscicida* pathogenesis.

## Introduction

*Edwardsiella piscicida* PPD130/91, previously known as *Edwardsiella tarda* PPD130/91 [1], is an intracellular bacterium, belonging to the family of Enterobacteriaceae. Based on phenotypic characterization, DNA-DNA hybridization and phylogenetic analysis, *E. tarda* strains were further classified into three species *E. tarda*, *E. piscicida* and * E. anguillarum* [1–3]*. E. tarda* infects humans and possesses neither the T3SS nor type VI secretion system (T6SS) gene cluster [1]; *E. piscicida* infects a wide range of fish species of both freshwater and marine, causing hemorrhagic septicemia mainly in Japanese flounder (*Paralichthys olivaceus*) and turbot (*Scophthalmus maximus*), and only one set of T3SS and T6SS gene cluster is distributed in its genome [1–4]; *E. anguillarum* infects eel, it has acquired the locus for enterocyte effacement (LEE) genes and contains 2 sets of T3SS and 3 sets of T6SS gene clusters [1].

*E. piscicida* is able to invade and replicate in epithelial and phagocytic cells, such as Epithelioma papillosum cyprinid (EPC) cells, HEp-2 cells, and murine macrophage J774A.1 cells [5–7]. The Type III secretion system (T3SS) is one of the two most important virulence factors in *E. piscicida* [6, 8]. T3SS is a membrane-spanning macromolecular machine that comprises more than 20 different proteins, through which effectors are delivered into host cells to cause infection [9]. Three translocon proteins (EseB, EseC and EseD) and two effector proteins (EseJ and EseG) are encoded within the *E. piscicida* T3SS gene cluster [6, 10, 11]. In the presence of host cells, EseB, EseC and EseD can form pores on the host membrane, through which effectors are translocated. In the absence of host cells, EseB forms filamentous appendages and mediates autoaggregation and biofilm formation in *E. piscicida* [12]. EsaN is an ATPase that energizes the transportation of T3SS substrates [10]. Deleting a translocon gene, such as *eseB*, *eseC*, *eseD* or an ATPase gene *esaN*, increases the 50% lethal dose (LD_50_) by approximately 1 log in blue gourami fish [6, 10]. EseJ inside *E. piscicida* inhibits its adhesion to EPC cells through negative regulation on type 1 fimbriae [11, 13]. *E. piscicida* type 1 fimbrial major subunit FimA is involved in its adhesion to fish epithelial cells [14–16].

The function of *orf1B* within T3SS gene cluster of *E. piscicida* remains obscure. Orf1B shares similarity with EscO of enteropathogenic *Escherichia coli* (EPEC), Spa13 of *Shigella flexneri*, SpaM/InvI of *Salmonella* SPI-1 (*Salmonella* Pathogenicity Island 1). Moreover, its structure resembles YscO of *Vibrio parahaemolyticus* and flagellar FliJ. EscO, encoded by a gene in the locus of enterocyte effacement (LEE) pathogenicity island, is essential for secretion of all categories of T3SS substrates in EPEC, moreover, EscO interacts with the ATPase EscN and stimulates EscN enzymatic activity [17]. Spa13 interacts with the ATPase Spa47, the C-ring protein Spa33, and the inner-membrane protein Spa40. Moreover, Spa13 can stabilize the needle protein MixH, thus influencing the secretion of type III proteins [18]. SpaM is required for efficient secretion of translocon proteins SipB and SipC and effector proteins SipA, SipD and SptP by *Salmonella* SPI-1 [19]. *Yersinia* YscO interacts with needle length control protein YcsP to control Yop secretion [20, 21], while YscO is not required for the assembly of the ATPase YscN [22]. FliJ of the *Salmonella enterica* serovar Typhimurium is remarkably similar to the γ subunit of FoF1-ATP synthase, FliJ promotes the formation of FliI (ATPase) hexamer rings by binding to the center of the ring [23, 24].

Orf1B was functionally characterized in this study, and it was revealed that Orf1B promotes the secretion of T3SS proteins probably by interacting with T3SS ATPase EsaN, contributing to its adhesion to epithelial cells by controlling the steady-state protein level of EseJ inside *E. piscicida.*

## Materials and methods

### Bacterial strains and culture

The bacterial strains and plasmids used in this study are listed in Table 1. *E. piscicida* PPD130/91 [25] were grown in tryptic soy broth (TSB, BD Biosciences) at 28 °C and *E. coli* strains in Luria–Bertani broth (LB, BD Biosciences) at 37 °C. To activate T3SS, *E. piscicida* strains were cultivated in Dulbecco modified Eagle medium (DMEM, Invitrogen) at 25 °C under a 5% (vol/vol) CO_2_ atmosphere. Antibiotics were supplemented at the following concentrations when required: 12.5 μg/mL colistin (Col; Sigma), 34 μg/mL chloramphenicol (Cm; Sigma), 50 μg/mL gentamicin (Gem; Sigma), and 50 μg/mL kanamycin (Km; Sigma).

**Table 1:** **Bacterial strains and plasmids used in this study.**

Strains or plasmids	Description and/or genotype	References or source
Strains		
*E. piscicida*		
WT	PPD130/91, Km^s^, Col^r^, Amp^s^	[25]
Δ*esaN*	In-frame deletion of aa 21–421, PPD130/91	[10]
Δ*orf1B*	In-frame deletion of aa 7–117, PPD130/91	This study
WT/pACYC184	WT strain transformed with pACYC184	This study
Δ*orf1B*/pACYC184	Δ*orf1B* strain transformed with pACYC184	This study
Δ*orf1B*/*orf1B*	Δ*orf1B* with pACYC184-HA-*orf1B*	This study
WT *esaN*::3 × FLAG	PPD130/91, with chromosomal expression of EsaN-3 × FLAG, Amp^r^, Km^r^	This study
WT *esaN*::3 × FLAG/*orf1B*	WT *esaN*::3 × FLAG with pACYC184-HA-*orf1B*	This study
WT *eseE*::3 × FLAG	PPD130/91, with chromosomal expression of EseE-3 × FLAG, Amp^r^, Km^r^	This study
WT *eseE*::3 × FLAG/*orf1B*	WT *eseE*::3 × FLAG with pACYC184-HA-*orf1B*	This study
Plasmids		
pACYC184	Tet^r^, Cm^r^	Amersham
pACYC184-HA-*orf1B*	pACYC184 with HA-*orf1B*	This study
pRE112	pGP704 suicide plasmid, *pir* dependent; Cm^r^ *oriT*, *oriV*, *sacB*	[27]
pKD46	Red helper plasmid, Amp^r^	[30]
pKD4	Template plasmid with FLP recognition target site, Km^r^	[30]

Superscripts—r, resistance; s, sensitivity.

Col, colistin; Amp, ampicillin; Tet, tetracycline; Km, kanamycin; Cm, chloramphenicol.

### Cell and culture condition

EPC cells [26] were grown at 28 °C in M199 medium (HyClone) supplemented with 10% heat-inactivated fetal bovine serum (FBS, Gibco) under a 5% (vol/vol) CO_2_ atmosphere.

### Construction of deletion mutants, complementation, and plasmids

Primers used in this study are listed in Table 2. Non-polar *orf1B* deletion mutants were generated by *sacB*-based allelic exchange [27]. Briefly, primer pairs *orf1B*-for/*orf1B*-int-rev, *orf1B*-int-for/*orf1B*-rev were used to generated an 845-bp fragment containing the upstream region of *orf1B*, and a 754-bp fragment containing the downstream region of *orf1B* from PPD130/91 genomic DNA. A 16 bp overlapping sequence introduced into the flanking DNA fragments permitted the fusion of the upstream and downstream regions together by a second PCR with primers *orf1B*-for and *orf1B*-rev. The resulting PCR product was ligated into the suicide vector pRE112 [27] before being transformed into *E. coli* S17-1 λpir. Through conjugation, a Δ*orf1B* strain was screened on 10% sucrose–tryptic soy agar plates and was verified by PCR and sequencing. The mutant strains show no defect in growth when cultured in TSB or DMEM.

**Table 2:** **Primers used in this study.**

Primers	Sequences (5′–3′)
*orf1B*-for	ATGGTACCCGCCTGGGCATCTTCTC
*orf1B*-int-rev	GCGTAGCTGCGTCAGCATGG
*orf1B*-int-for	GCTGACGCAGCTACGCCTGGAGGAGAGCGAATGA
*orf1B*-rev	ATGGTACCGCAGCCACGCATTCAGC
*orf1B*-com-for	CACTGTCCGACCGCTTTGGCCGGGTGGGCGAATACCGAGC
*orf1B*-com-int-rev	ATATCGCGTAGCTGCGTCAGAGCGTAATCTGGAACATCGTATGGGTACATGGCTGGCCT
*orf1B*-com-int-for	AGGCCAGCCATGTACCCATACGATGTTCCAGATTACGCTCTGACGCAGCTACGCGATAT
*orf1B*-com-rev	GCCAAAGCGGTCGGACAGTGCTCCGAGA
pACYC184-for	GGCCTCAACCTACTACTGGGCTGCTTCC
pACYC184-rev RT-16S-for RT-16S-rev RT-*eseJ*-for RT-*eseJ*-rev	GCCAAAGCGGTCGGACAGTGCTCCGAGA ACTGAGACACGGTCCAGACTCCTAC TTAACGTTCACACCTTCCTCCCTAC CTGCTGGCGACCTTTTATCC AGTAGTCCGGATTGCGCTTA
*esaN*::3 × FLAG-for	GCCACCCTCAGCGCACTGTACAGCGCCGTAGGCCAGCCATGCGACTACAAAGACCATGACGGTGATTATAAAGATCATGACATCGACTACAAG
*esaN*::3 × FLAG-rev	CTGGAGCTGCTGCTGTCGTCGCCGTACGCCGTCAGCCTCATC
*eseE*::3 × FLAG-for	ATCGTCTGGATGATGCGCTGTGGCTGCGCTATTGCTGCCGCGACTACAAAGACCATGACGGTGATTATAAAGATCATGACATCGACTACAAG
*eseE*::3 × FLAG-rev	GATATATTATTACATTATCAGGGCGTGCGCCCCGGGGCTTTGGAAACATATGAATATCCTCCTTAGT

To obtain the DNA sequence covering ribosome binding site, hemagglutinin tag (HA tag, sequence: YPYDVPDYA) and *orf1B* gene, two PCR fragments were first obtained from PPD130/91 genomic DNA by primer pairs *orf1B*-com-for plus *orf1B*-com-int-rev, *orf1B*-com-int-for plus *orf1B*-com-rev, which were overlapped with the primer pair of *orf1B*-com-for plus *orf1B*-com-rev before being inserted into pACYC184 (Amersham). The pACYC184-HA-*orf1B* obtained was introduced into the Δ*orf1B* strain to obtain the Δ*orf1B/orf1B* strain; expression of HA-Orf1B was confirmed by immunoblotting with mouse anti-HA antibody.

### Adhesion assay by confocal microscopy

Adhesion assay was performed as described by Xie et al. [11]. Briefly, EPC monolayers were washed once with pre-warmed M199 medium (28 °C) and infected at a multiplicity of infection (MOI) of 10 with *E. piscicida* PPD130/91 strains wt/pACYC184, Δ*orf1B*/pACYC184, and Δ*orf1B*/*orf1B* (Table 1). After 30 min of infection, the EPC monolayers were washed three times and fixed in 4% paraformaldehyde (PFA) and stained with mouse anti-LPS antibody and Alex 488 Goat anti-mouse IgG (Invitrogen). The adherence rates of each infection condition were examined from two wells in triplicate experiments. Fourteen images were photographed randomly from two replicates under a confocal laser scanning microscope (Leica SP8). Representative images and mean ± standard deviations (SD) from one representative experiment were shown. Student’s *t* test was used to analyse the difference between the two groups.

To prepare mouse anti-LPS antibody, LPS was isolated according to the manufacturer’s protocol (Bestbio) from 30 mL *E. piscicida* PPD130/91 cultured in TSB, and the LPS antibody was prepared as described by Gao et al. in eight 6-week-old naïve C57BL/6 mice by the company of Daian, China [12].

### Quantitative reverse transcription-PCR

Overnight cultures of *E. piscicida* WT strain, Δ*orf1B* strain, and Δ*orf1B*/*orf1B* strain were subcultured at 1:100 into 10 mL DMEM and grown at 25 °C under a 5% (vol/vol) CO_2_ atmosphere for 24 h. Bacteria from three replicates of each strain were harvested. Total RNA was isolated according to the manufacturer’s protocol with the RNeasy Mini Kit (Qiagen), followed by DNase I treatment for the elimination of genomic DNA contaminants. Upon the RNA reverse transcription, nine cDNA libraries (WT-1, WT-2, WT-3, Δ*orf1B*-1, Δ*orf1B*-2, Δ*orf1B*-3, Δ*orf1B*/*orf1B*-1, Δ*orf1B*/*orf1B*-2, and Δ*orf1B*/*orf1B*-3) were obtained. Real-time PCR was performed on a CFX96 real-time system (Bio-Rad) using the SYBR green reagent. The qPCR was run at 95 °C for 10 min, followed by 40 cycles of 95 °C for 15 s, 58 °C for 20 s, 72 °C for 30 s, and 75.5 °C for 5 s. The mRNA expression level was normalized against the level of 16S rRNA gene expression. The relative transcriptional level of *eseJ* in the Δ*orf1B* and Δ*orf1B*/*orf1B* strains compared to that in the WT was then calculated using the formula 2^−ΔΔCT^, where CT is the threshold cycle and ΔΔC_T_ is equal to the change in the C_T_ (ΔC_T_) for the Δ*orf1B* or Δ*orf1B*/*orf1B* strain minus ΔC_T_ for the wild type. The *t* test implemented in SPSS was used to calculate the *P* value with a hypothetical mean of 1.0. The data shown are the means ± SD from triplicate samples.

### Expression and secretion assay

Overnight cultures of *E. piscicida* strains were subcultured at 1:100 into DMEM and grown static at 25 °C for 24 h. The total bacterial proteins (TBP) and the extracellular proteins (ECP) were prepared as described by Zheng and Leung [8] before being subjected to immunoblotting with mouse antibodies against DnaK at 1:2000 (Stressgen), and with rabbit antibodies against EseJ at 1:2000 [11], EseG at 1:2000 [10], EseB at 1:2000 [28], EseC at 1:2000 [29], EseD at 1:2000 [29] and EvpC at 1:2000 [8]. Horseradish peroxidase (HRP)-conjugated goat anti-mouse IgG or Goat anti-rabbit IgG (Millipore) were used at a dilution of 1:5000.

### Co-immunoprecipitation (Co-IP) assay

The chromosomal copy of *esaN* or *eseE* in *E. piscicida* was tagged with the 3 × FLAG epitope by the Red recombination system [30]. pACYC184-HA-*orf1B* was introduced into each constructed strain. The WT *esaN*::3 × FLAG/*orf1B* and WT *eseE*::3 × FLAG/*orf1B* strains were verified by immunoblotting with anti-FLAG antibody. Bacterial pellet from 20 mL culture of WT *esaN*::3 × FLAG/*orf1B* or WT *eseE*::3 × FLAG/*orf1B* strain was resuspended with phosphate buffered saline (PBS) supplemented with 1.0 mM phenylmethanesulfonyl fluoride (PMSF). After sonification, the cell lysates were centrifuged at 16 000*g* for 10 min and the supernatants were mixed with NP-40 at a final concentration of 1% for 30 min before being pre-cleared with protein G-immobilized beads (Thermo) for 1 h at 4 °C. The pre-cleared cell lysates were precipitated with anti-HA antibody for 4 h before being incubated with protein G immobilized beads overnight. The beads were washed four times before being resuspended in 1 × SDS sample buffer, and analyzed by immunoblotting against HA tag and FLAG tag.

### Single-strain infection in blue gourami fish

Single-strain infection in naïve blue gourami fish [*Trichogaster trichopterus* (Pallas)] (11.92 ± 1.62 g) was performed to investigate the contribution of Orf1B in pathogenesis of *E. piscicida*. Healthy blue gourami fish were maintained in 25 ± 0.5 °C. The *E. piscicida* WT and Δ*orf1B* strains were subcultured at 1:20 into TSB overnight and grown at 28 °C for 3 h. The bacteria were washed three times with PBS, and the OD_540_ values were adjusted to 0.5. Equal amounts of bacteria were injected intramuscularly (i.m.) at 4.5 × 10^5^ CFU into each fish, using 20 fish per group. The experiment was performed independently three times, and one representative data are shown. Differences in fish survival were assessed using the Long-rank (Mantel–Cox) test.

The experiments with fish were performed in strict accordance with the recommendations in the Guide for the Care and Use of Laboratory Animals of the Chinese Academy of Sciences. The protocol was approved by the Ethics Committee on Animal Experiments of the Institute of Hydrobiology (Permit Number E0110305).

## Results

### Sequence analysis of Orf1B and EsaN

**Figure 1:**
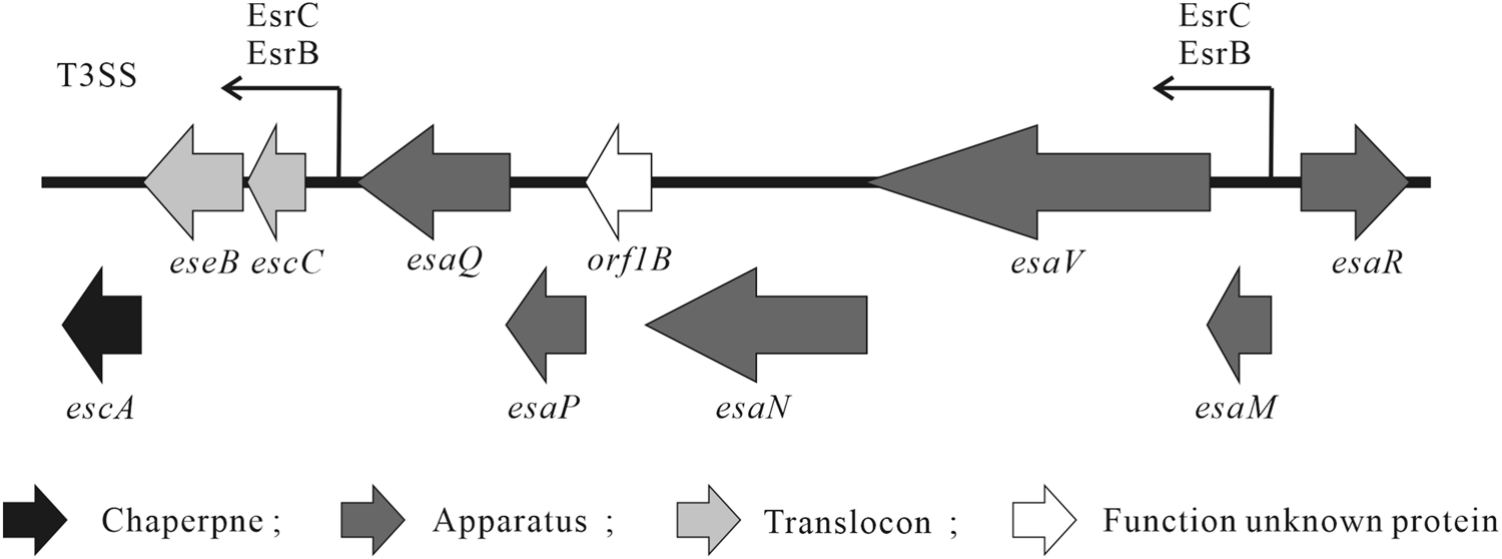
**Schematic representation of the *escA*-*esaR* region of *E. piscicida* T3SS.** Arrows represent each of the open reading frames, the bent arrow represents the putative promoter region.

The *orf1B* gene is located upstream of *esaP* and *esaQ* and downstream of *esaN*, *esaV*, and *esaM* in the *E. piscicida* T3SS gene cluster (Figure 1). It encodes a 14.20 kDa protein with pI of 8.04. Using the SWISS-MODEL analysis [31], Orf1B was shown to structurally share 13.45% identity with a putative type III protein YscO from *V. parahaemolyticus*, and 9.09% identity with FliJ protein, a flagellar type III export apparatus from *S. enterica* serovar Typhimurium. Similarity analysis on Orf1B and EsaN revealed that Orf1B protein shares similarity with EscO of enteropathogenic *Escherichia coli* (EPEC), Spa13 of *Shigella flexneri*, SpaM/InvI of *Salmonella* SPI-1, and YscO of *Yersinia pseudotuberculosis* by 26.4%, 24.1%, 20.8%, and 32.5%, respectively, while T3SS ATPase EsaN shares similarity with InvC of *Salmonella*, Spa47 of *Shigella*, YscN of *Yersinia*, and EscN of EPEC by 41.6%, 43.4%, 53.5%, and 49.3%, respectively (Table 3).

**Table 3:** **Similarity matrix for Orf1B and EsaN.**

Protein	Homologous protein (% similarity)			
*E. piscicida*	*Salmonella*	*Shigella*	*Yersinia*	EPEC
Orf1B	SpaM (20.8%)	Spa13 (24.1%)	YscO (32.5%)	EscO (26.4%)
EsaN	InvC (41.6%)	Spa47 (43.4%)	YscN (53.5%)	EscN (49.3%)

The percentage of similar amino acid residues was calculated using MegAlign program within the DNASTAR package (DNASTAR, Madsion, USA). The corresponding protein sequences are retrieved through the following Genbank accession numbers: Orf1B (WP_034171802.1); SpaM (WP_001638823.1); Spa13 (WP_024260642.1); YscO (WP_002212952.1); EscO (WP_209292174.1); EsaN (AAX55238.1); InvC (AAA74038.1); Spa47 (AAP79012.1); YscN (AAN37557.1), and EscN (AAL57542.1).

### Orf1B plays a pivotal role in T3SS protein secretion in *E. piscicida*

**Figure 2:**
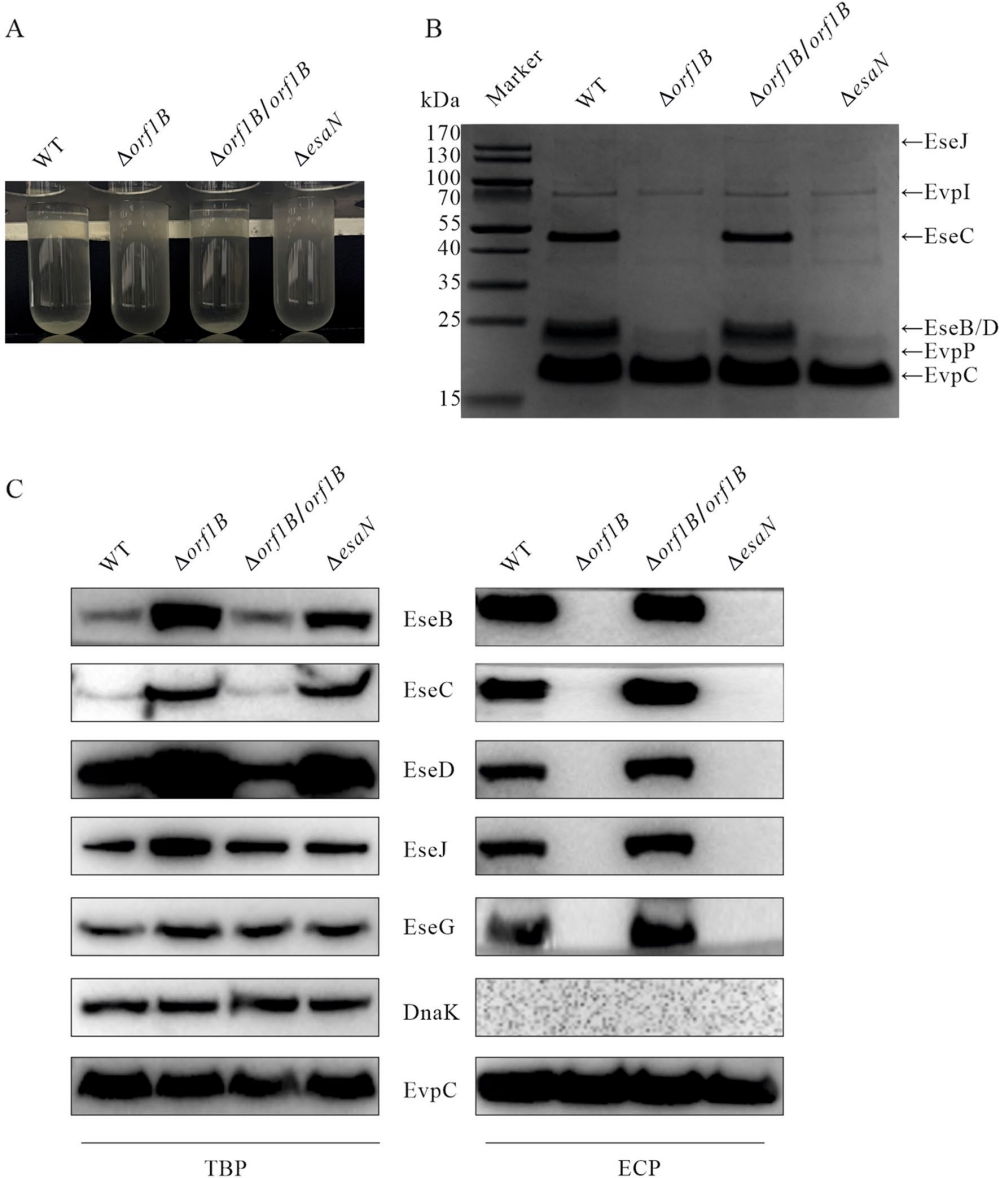
**Orf1B is required for efficient secretion of EseJ, EseG, EseB, EseC, and EseD. A** Autoaggregation of *E. piscicida* strains at 24 h post-subculture into DMEM. **B** Secretion profiles of *E. piscicida* strains. Samples of ECP from similar amounts of *E. piscicida* strains grown in DMEM were separated using SDS-PAGE gel and stained with Coomassie blue. EseJ, EseC, EseB, and EseD are T3SS proteins, EvpI, EvpP, and EvpC are T6SS proteins. **C** Orf1B is required for efficient secretion of T3SS translocon proteins and effectors. Total bacterial proteins (TBP; left panel) and extracellular proteins (ECP; right panel) from similar amounts of the WT strain, Δ*orf1B* strain, Δ*orf1B/orf1B* strain and Δ*esaN* strain were probed with EseB, EseC, EseD, EseG, EseJ, DnaK, and EvpC antibodies. EvpC, a protein secreted by T6SS but not by T3SS, was used as a loading control. The immunoblotting data shown are representative of three independent experiments.

To explore the phenotype of Orf1B protein, *E. piscicida* WT strain, Δ*orf1B* strain, Δ*orf1B/orf1B* strain, and Δ*esaN* strain were subcultured into DMEM and the autoaggregation of each strain was compared at 24 h post-subculture. EsaN is an ATPase that energizes the transportation of T3SS substrates [10]. As shown in Figure 2A, the culture of WT and Δ*orf1B*/*orf1B* strain settled to the bottom of the glass tubes, and their supernatants became clear, however, the culture of the Δ*orf1B* and Δ*esaN* strains remained cloudy. This indicates that disruption of Orf1B abolishes its autoaggregation. Autoaggregation of *E. piscicida* in DMEM is mediated by its T3SS translocon protein EseB [12]. To investigate whether deletion of *orf1B* influences the secretion of EseB, the culture supernatants of the four *E. piscicida* strains aforementioned were collected and their extracellular protein profiles were compared on an SDS-PAGE gel stained with Coomassie blue. As shown in Figure 2B, neither T3SS translocon proteins EseB, EseC, EseD nor T3SS effector EseJ was secreted from Δ*orf1B* or Δ*esaN* strains; complementation of Δ*orf1B* strain with pACYC184-HA-*orf1B* restored their secretion to the level of the wild-type strain.

Does deletion of *orf1B* influence the steady-state protein levels of T3SS translocon and effectors? To address this question, similar amounts of bacterial pellets (TBP) and extracellular proteins (ECP) from those four strains were immunoblotted. EvpC, a major protein secreted via T6SS but not T3SS was used as a loading control [8]. Increased protein levels of T3SS translocon EseB/EseC/EseD and effector EseJ were observed in TBP of Δ*orf1B* strain or Δ*esaN* strain compared to that in the WT strain. Complementation of Δ*orf1B* strain with pACYC184-HA-*orf1B* restored T3SS translocon and effectors to the levels of the wild-type strain (Figure 2C, left panel). Neither T3SS translocon EseB/EseC/EseD nor T3SS effectors EseG and EseJ were secreted from Δ*orf1B* strain or Δ*esaN* strain (Figure 2C, right panel). DnaK is a bacterial cytosolic marker. DnaK was not detected in any ECP, showing that detection of T3SS translocon and effectors is not due to leakage from bacterial pellets. Taken together, these data indicate that, like EsaN, Orf1B is necessary for the activity of *E. piscicida* T3SS, and disruption of Orf1B blocks the secretion of T3SS translocon and effectors, resulting in their accumulation inside *E. piscicida*.

### Orf1B interacts with EsaN as revealed by co-immunoprecipitation

**Figure 3:**
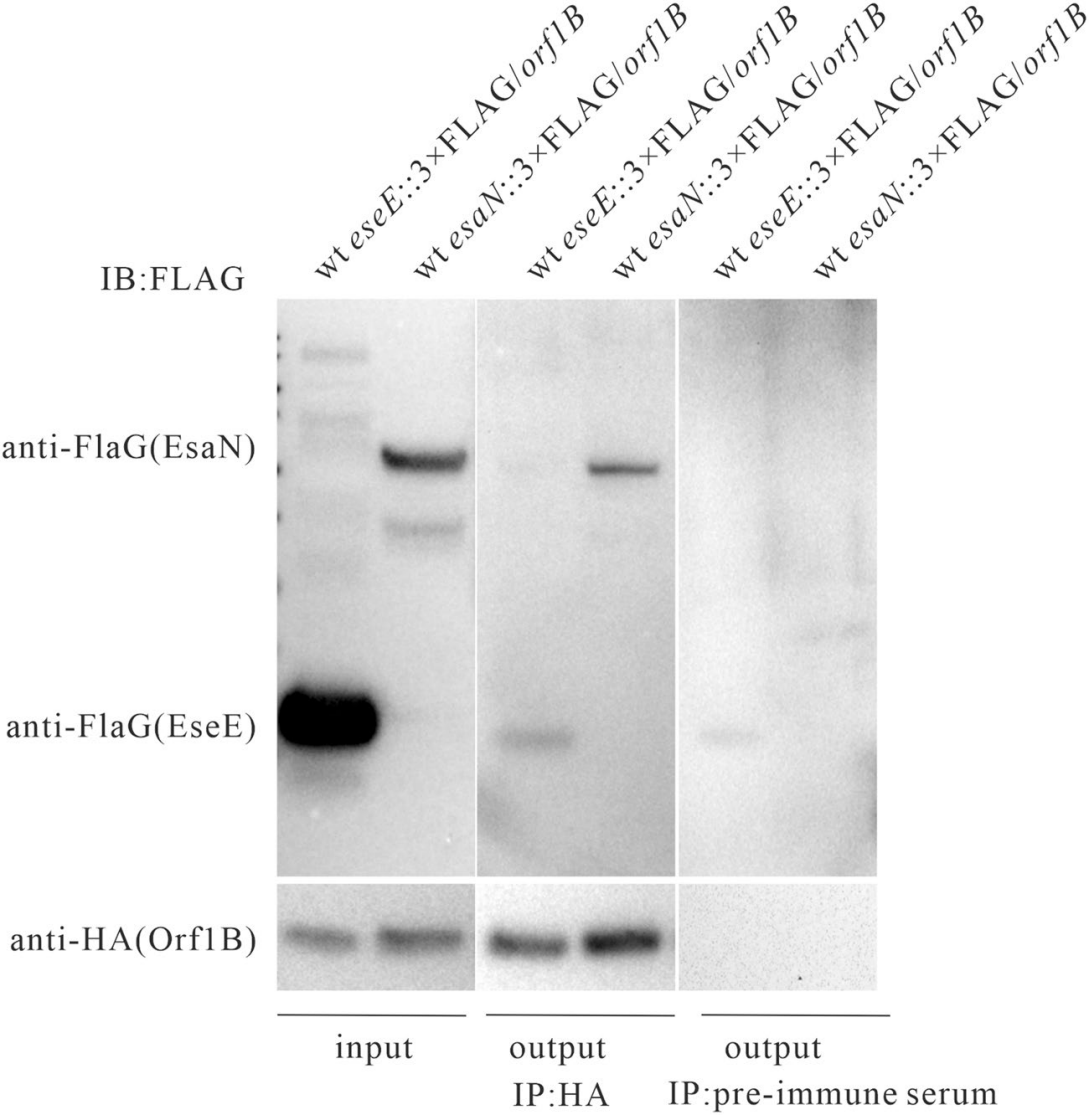
**Orf1B interacts with EsaN.** WT *esaN*::3 × FLAG/*orf1B* and WT *eseE*::3 × FLAG/*orf1B* strains cultured in DMEM were harvested and sonicated to produce cell lysates. These cell lysates were immunoprecipitated (IP) with an anti-HA antibody (rabbit, left panel), or with the pre-immune serum from rabbit (as a control). HA-Orf1B protein was detected by the anti-HA antibody, EsaN-3 × FLAG or EseE-3 × FLAG was detected with an anti-FLAG antibody. The band of EseE-3 × FLAG in the output of the left panel is non-specific as it was also detected from the IP with the pre-immune rabbit serum (right panel).

Considering that several homologues of Orf1B interact with ATPase [17–19, 24], the interaction between Orf1B and the T3SS ATPase EsaN was investigated through immunoprecipitation. WT *esaN*::3 × FLAG/*orf1B* and WT *eseE*::3 × FLAG/*orf1B* strains cultured in DMEM were harvested and sonicated to produce cell lysates. These cell lysates were immunoprecipitated (IP) with an anti-HA antibody (rabbit, left panel). In parallel, we performed co-IP with the pre-immune serum from rabbits (as a control, right panel) (Figure 3). HA-Orf1B protein was detected by the anti-HA antibody, EsaN-3 × FLAG or EseE-3 × FLAG were detected with an anti-FLAG antibody. It was observed that EsaN-3 × FLAG was precipitated by HA-Orf1B, while EseE-3 × FLAG was also precipitated by HA-Orf1B. Meanwhile, the interaction of HA-Orf1B with EseE-3 × FLAG is non-specific, as co-IP with the pre-immune serum of rabbit also detected this interaction. This is probably due to the overwhelming protein level of EseE inside *E. piscicida*. Taken together, Orf1B specifically interacts with EsaN in *E. piscicida*.

### Orf1B contributes to *E. piscicida* adhesion to EPC cells

**Figure 4:**
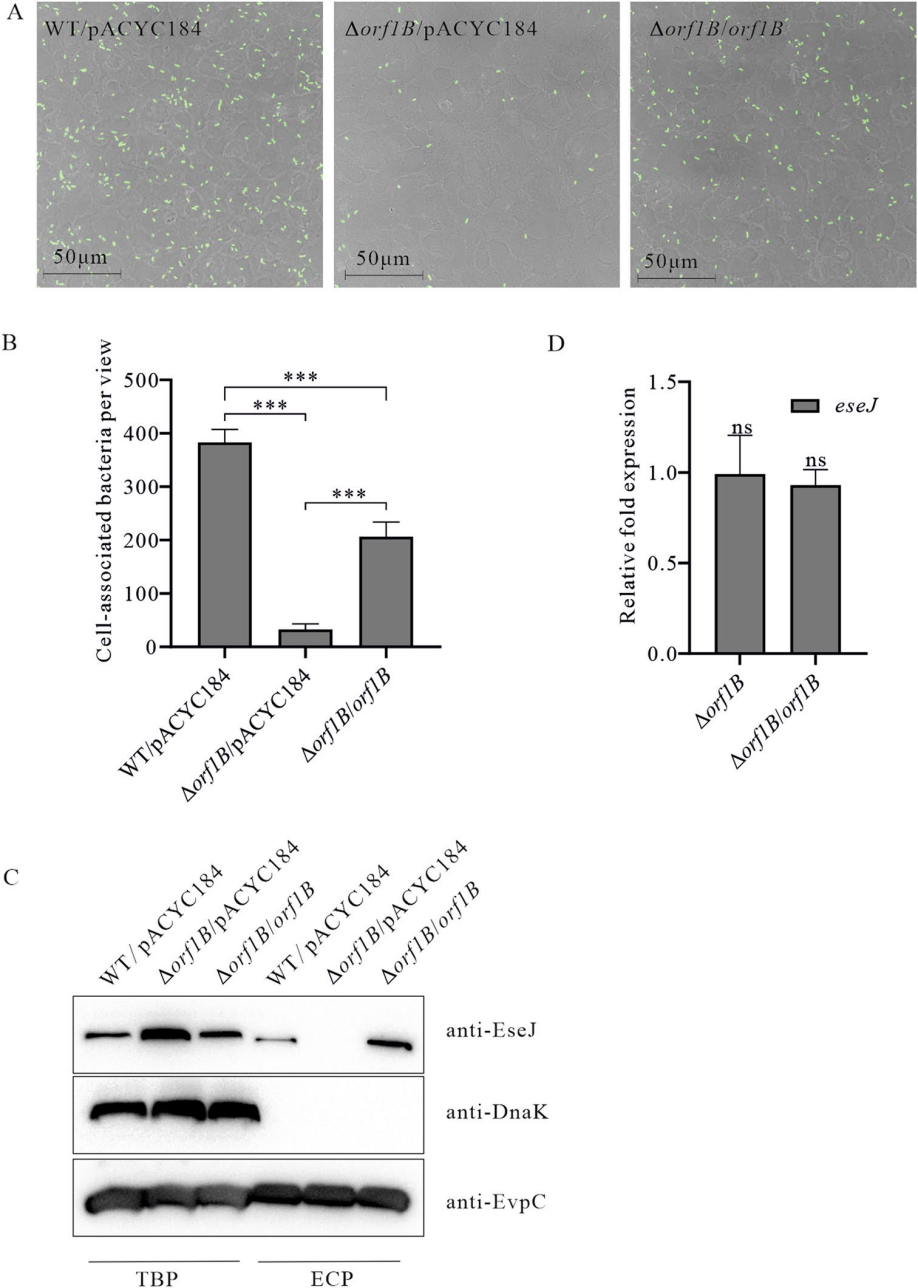
**Orf1B contributes to *E. piscicida* adhesion to fish EPC cells by facilitating secretion of EseJ. A** Representative images of infected EPC cells. Fish EPC cells were infected at an MOI of 10. After a 30-min incubation, cells were fixed and stained with anti-LPS (*E. piscicida*) antibody and Alex 488 Goat anti-mouse antibody before being imaged under confocal microscope. Bars, 50 μm. **B** Quantification of cell-associated bacteria per view. Fourteen views from each infection were quantified, and the mean ± SD from one representative experiment are shown. ***P < 0.001. **C** Increased steady-state protein level of EseJ upon disruption of Orf1B. Total bacterial proteins (TBP) and extracellular proteins (ECP) from similar amounts of WT strain, Δ*orf1B* strain, and Δ*orf1B/orf1B* strain cultured in TSB were probed with anti-EseJ and anti-EvpC antibodies. EvpC, a protein secreted by T6SS but not by T3SS, was used as a loading control. The immunoblotting shown are representative of three independent experiments. **D** The transcription level of *eseJ* was not interfered by the disruption of Orf1B. The mRNA levels of *eseJ* in wild-type strain, Δ*orf1B* strain, and Δ*orf1B/orf1B* strain were examined by qRT-PCR. Gene expression levels in the Δ*orf1B* strain, and Δ*orf1B/orf1B* strain relative to the level in the wild-type strain are presented as relative fold changes in gene expression. 16S rRNA was used as the reference gene. Data are presented as means ± SD. The *t* test implemented in SPSS was used to calculate the *P* value with a hypothetical mean of 1.0. NS, not significantly different.

*Edwardsiella piscicida* is able to adhere to and invade EPC cells [6]. To investigate whether or not Orf1B plays a role in this process, EPC cells were infected with the WT strain, Δ*orf1B* strain and Δ*orf1B/orf1B* strain and cell-associated *E. piscicida* strains were quantified. It was found that the cell-associated Δ*orf1B* strain was 11 ± 0.74 folds less than that of the wild-type strain. Complementation of the Δ*orf1B* strain partially increased its adhesion to the level of the wild-type strain (Figure 4A and B). This demonstrates that Orf1B contributes to *E. piscicida* adhesion to EPC cells.

T3SS protein EseJ negatively regulates *E. piscicida* adherence to EPC cells from within bacteria [11]. By immunoblotting on TBP and ECP of the three strains, it was observed that the steady-state protein level of EseJ in the Δ*orf1B* strain was much higher than that in the WT strain or Δ*orf1B/orf1B* strain when cultured in TSB, and no EseJ was secreted by Δ*orf1B* strain (Figure 4C). EvpC, a protein secreted via T6SS, was used as a protein loading control. The cytoplasmic protein DnaK was not detected in any ECP, suggesting that the EseJ that was detected from the ECP of WT strain or Δ*orf1B/orf1B* strain was not due to leakage from the bacterial pellets. To investigate whether or not Orf1B regulates *eseJ*, the transcription levels of *eseJ* in the WT, Δ*orf1B* stran, and Δ*orf1B/orf1B* strains were compared. As shown in Figure 4D, no significant difference between each strain in *eseJ* transcription was detected. Taken together, these data indicate that the EseJ protein accumulated inside the Δ*orf1B* strain because its secretion blockage led to the decreased adhesion of *E. piscicida* to EPC cells.

### Orf1B contributes to *E. piscicida* pathogenesis in vivo

**Figure 5:**
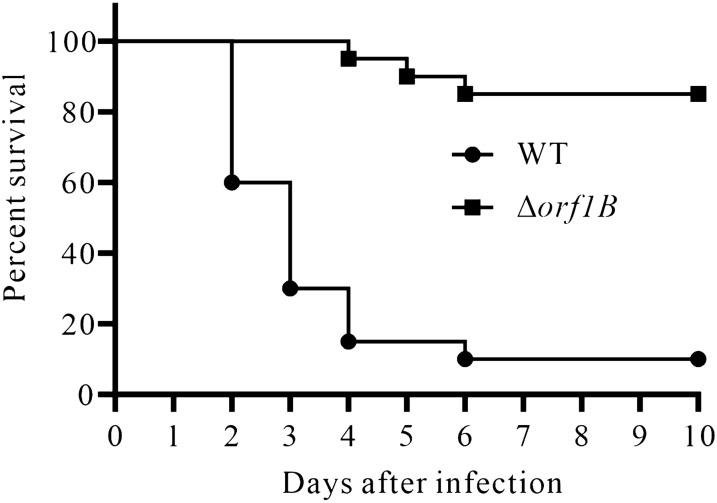
**Survival curve of blue gourami fish infected with *E. piscicida* wild-type strain (WT) and Δ*orf1B* strain.** Fish survival was determined for a period of 10 days. Each group of 20 fish were injected at a dosage of 4.5 × 10^5^ CFU. The result of one representative experiment was presented. Significant difference in fish survival curve was assessed using the Two-tailed long-rank (Mantel–Cox) test, *P* < 0.001.

To determine the contribution of Orf1B in the pathogenesis of *E. piscicida*, the survival rates of blue gourami fish were assessed following bacterial infections. Each blue gourami fish was injected with 4.5 × 10^5^ CFU of *E. piscicida*. Ten days post-infection, 10% of the fish infected with the wild-type strain (WT) survived, whereas 85% of the fish infected with *E. piscicida* Δ*orf1B* strain survived. Of note, the blue gourami infected with WT strain began to die at 2 days post infection, and the death rate increased sharply between day 2 and day 4, while a low death rate occurred when infected with the Δ*orf1B* strain. A significant difference (*P* < 0.001) was detected between the survival curve of blue gourami fish infected with the WT and Δ*orf1B* strains (Figure 5). This indicates that deletion of *orf1B* significantly attenuates the virulence *of E. piscicida* PPD130/91 in vivo.

## Discussion

Bacterial type III secretion machines are used to translocate effector proteins from the bacterial cytosol directly into eukaryotic cells [9], and secretion of T3SS translocon and effectors are tightly controlled [32–34]. In this study, we demonstrate that Orf1B controls secretion of T3SS translocon and effectors by interacting with ATPase protein EsaN, and Orf1B contributes to *E. piscicida* adhesion through facilitating the secretion of EseJ.

The autoaggregation of *E. picicida* cultured in DMEM was abolished in either Δ*orf1B* strain or Δ*esaN* strain because of their failure in EseB secretion. Secreted EseB forms filaments on the surface of *E. piscicida*, and the connection between EseB filaments among different bacteria mediates autoaggregation and biofilm formation in *E. piscicida* [12]. Besides EseB, neither the T3SS translocon protein EseC/EseD nor T3SS effector EseJ could be secreted by the Δ*orf1B* strain; conversely, increased steady-state protein levels of EseB/EseC/EseD and EseJ were detected inside the Δ*orf1B* strain when compared with that in the WT strain. Similar protein profiles were observed in the Δ*esaN* strain as in the Δ*orf1B* strain. This leads us to speculate that Orf1B either functions as an apparatus or accompanies T3SS ATPase EsaN.

Disruption of Orf1B leads to the failure of T3SS protein secretion in *E. piscicida*, this is similar to the phenotypes of its homolog EscO, Spa13, YscO, SpaM, and FliJ [17–19, 24]. EscN of EPEC forms an asymmetric ring, and the T3SS inner stalk protein EscO colied coli interacts with EscN at its C-terminal domain, which stimulates its ATP hydrolytic activity, energizing T3SS protein secretion [35, 36]. Consistently, Spa13 interacts with the ATPase Spa47 in *Shigella flexneri* [18]. However, *Yersinia* YscO is not required for the assembly of ATPase YscN [22], YscO interacts with chaperone SycD, and its interaction with the needle length control protein YscP is essential to type III protein secretion [35]. In this study, we provide evidence that Orf1B interacts with T3SS ATPase EsaN, it is speculated that by stimulating ATP hydrolytic activity of EsaN, Orf1B contributes to the secretion of T3SS proteins in *E. piscicida*.

Adhesion of bacteria to interfaces is the first step in pathogenic infection, and bacteria use a variety of surface structures to promote interfacial adhesion. Yfco fimbriae enhances adherence and colonization of avian pathogenic *Escherichia coli* (APEC) in vivo and in vitro [37]. Fimbriation level alters the adhesion of *Escherichia coli* to interfaces [38]. Type 1 fimbria plays a pivotal role in *E. piscicida* adhesion to epithelial cells [13–16]. Through suppression of type 1 fimbriae, T3SS protein EseJ suppresses *E. piscicida*’s adhesion to host epithelial cells [13]. In this study, decreased adhesion was observed for Δ*orf1B* strain when compared with WT strain. Based on the facts that the transcription level of *eseJ* did not change when Orf1B was disrupted, and that a higher steady-state protein level of EseJ was detected in the Δ*orf1B* strain than in the WT strain, it was concluded that Orf1B promotes *E. piscicida* adhesion by facilitating EseJ secretion.

In summary, we have demonstrated that Orf1B interacts with T3SS ATPase protein EsaN. This interaction may provide energy for the secretion of T3SS translocon and effectors, thus contributing to *E. piscicida* adhesion and pathogenesis.

## Data Availability

All data generated or analyzed in this study were included in this article.
